# New Studies of the Aberrant Alterations in Fibrillin-1 Methylation During Colorectal Cancer Development

**DOI:** 10.3389/fonc.2022.862887

**Published:** 2022-04-20

**Authors:** Ling Lv, Jianzhong Ma, Lina Wu, Chao Zhang, Yueping Wang, Guang Wang

**Affiliations:** ^1^ Department of Thoracic Surgery, The First Affiliated Hospital of China Medical University, Shenyang, China; ^2^ School of Business, Xianda College of Economics & Humanities, Shanghai International Studies University, Shanghai, China; ^3^ Department of Medical Laboratory, Shengjing Hospital of China Medical University, Shenyang, China; ^4^ Department of Infection Diseases, The First Affiliated Hospital of Hainan Medical University, Haikou, China; ^5^ Hepatobiliary Surgery Department, The First Affiliated Hospital, China Medical University, Shenyang, China

**Keywords:** methylation variation, prevention and diagnosis, epigenetics, biomarker, DNA methyltransferase

## Abstract

**Background:**

Fibrillin-1 (FBN1) methylation risk from control to colorectal cancer (CRC), the variation regularities of FBN1 methylation, and DNA methyltransferase (DNMT) catalyzed with FBN1 methylation had not been reported yet; these were all studied in this paper.

**Methods:**

FBN1 methylation roles were investigated with big data and meta-analysis.

**Results:**

The 6 independent studies were searched including 702 tissue and 448 feces. FBN1 methylation frequencies of CRC, adenoma or polyp, and control in tissue were 79.1%, 69.4%, and 2.7%, respectively; those in feces were 74.6%, 50.7%, and 10.8%, respectively. FBN1 methylation of control samples was used as a standard reference; this study showed that ORs (95% CI) of FBN1 methylation in CRC and control tissues were 124.79 (62.86–248.35); those in feces were detected to be 30.87 (16.48–57.85). FBN1 methylation risk in tissue was higher than that in feces; there was a quadratic equation between the methylation rate of tissue and that of feces. There was another quadratic curve in the variation process of FBN1 methylation; this curve reflected the overall metabolism regularity of DNMT.

**Conclusions:**

The transcriptional inactivation of FBN1 gene might start from normal colonic epithelium; the quadratic curve of FBN1 methylation catalyzed by DNMT can gradually produce powerful strength, accelerate expansion, and eventually lead to CRC. The overall metabolism regularity of DNMT maintains the changing process of FBN1 methylation; it has the changing feature of the same quadratic curve. FBN1 methylation is a promising biomarker. FBN1 methylation risk size in feces reflects that in tissue in non-invasive detection.

## Introduction

Epigenetic alteration is common in cancer occurrence and progression; DNA methylation is an important component of epigenetics. The epigenetic pathway of CpG island methylator phenotype (CIMP) had been used clinically in the diagnosis and screening of colorectal cancer (CRC), but the molecular pathological mechanism of CIMP is still not very clear. DNA methylation affected the expression of genes by interacting with the transcription factors or by changing the chromatin structure. Epigenetic regulators of gene expression were mainly the methylation of CpG islands, histone post-translational modifications (PTMs), and microRNAs (miRNAs) (1). Another reason was that aberrant DNA methylation at the 5-position of cytosine was catalyzed and maintained by DNA methyltransferase (DNMT), and it was associated with not only various cancers by silencing of tumor suppressor genes but also other diseases (2). The higher expression of DNMT was demonstrated in a variety of human malignancies, and tumor progression was facilitated by DNMT-mediated gene inactivation (3). Therefore, it is very important for the aberrant regulation of DNA methylation to be explored regarding gene expression, mediation, DNMT, and tumor development process. We all knew that the process of methylation changing was closely linked to the process of DNMT. During cancer occurrence and development, how did the overall changing process of DNA methylation affect gene expression? How did the overall changing process of DNMT catalyze and maintain DAN methylation and synthesize or degrade physiological activity? These problems were discussed with human methylation experiment data in this paper.

CRC is a common malignancy in the digestive system; its incidence and mortality rose worldwide (4), ranking third in malignant tumors (5). In western developed countries, the CRC mortality rate was 33%, and it is one of the most common causes of malignant tumors. The lifetime risk of CRC is as high as 5% in the American population (6). CRC had the characteristics of hidden onset, long course, and good early diagnosis and prognosis, which makes it suitable for screening. Numerous studies had shown that early screening diagnosis had reduced the incidence and mortality of CRC. The main aim of CRC screening was to find early tumors that were treatable or precancerous lesions that were highly likely to develop into malignant tumors. Colonoscopy was an important way to detect early CRC. However, as an invasive examination requiring complete and thorough intestinal preparation and other shortcomings, it is difficult to be widely accepted. The fecal occult blood testing (FOBT) with the non-invasive method was the current clinically recommended standard for early screening of CRC. However, its sensitivity was poor, generally less than 30%; it cannot meet the requirements of early diagnosis of CRC. Recent studies had shown that DNA methylation changes were closely related to the development of CRC (7) and that they run through the whole CRC development process. Second, the changes in abnormal DNA methylation often occur in the early stage of CRC. Therefore, searching for larger samples became a hot topic in order to find out reliable and good molecular biomarkers of CRC. Previous studies had identified fibrillin-1 (FBN1) as a potential optimal biomarker for early detection of CRC in relatively small population samples (8). However, the role and mechanism changing laws of FBN1 methylation had not been reported using relatively large sample data, for example, meta-analysis. In the present work, our study aimed to find out both the variation regularities and mechanisms changing the characteristics of FBN1 methylation and the relationship between FBN1 methylation in tissue colorectal cell and that in cell-free DNA feces during CRC tumorigenesis and to investigate whether FBN1 methylation acted as an early biomarker in screening of early CRC.

## Materials and Methods

The databases PubMed, Web of Science, CBA, BENDIPubmed, EMBASE, CNKI, and BAICHAIN had been searched using the systematic search method. The combination keywords were composed of FBN1, fibrillin-1, hypermethylation, methylation, adenoma, polyp, CRC, colorectal cancer, control, normal, tissue, and feces. The systematic search ended on August 4, 2021. The selected articles were also searched manually to identify other relevant independent studies. All published literature was collected in both English and Chinese languages. Based on our discussion, the independent literature that was involved in FBN1 methylation of the case–control study was identified and gathered.

Independent literature that had been searched must satisfy the following criteria: 1) the methods of FBN1 methylation experiment were shown according to methylation-specific PCR (MSP), bisulfite sequencing PCR (BSP), quantitative MSP (qMSP), and other methylation experiment methods. 2) Every literature must possess the study sample size and case–control study in tissue or feces detections. 3) The literature identified had different author names and independent samples. 4) The literature included the first author, publication year, and clinical outcomes. 5) If the data set is published in more than one literature, then only that of the most reasonable literature is included. According to the data extraction criteria above, the disagreement problems were resolved after discussions. Stepwise selection and elimination were run, and a total of 6 independent studies were ultimately received. The data set of FBN1 methylation incidences is summarized in **Table 1** (9–14). The separate information about control persons, adenomas and polyps, and carcinomas in both tissue and feces is provided in **Table 1**.

**Table 1:** The main characteristics and data of some references.

Ref	Author	Nationality	Year	Total	CRC	AP	control	Resource	Detection
(9)	Guro E	Norway	2011	446	142/179	77/111	3/156	Tissue	qMSP
(10)	Qi Guo	China	2013	150	59/75		3/75#	Tissue	MSP
(10)	Qi Guo	China	2013	105	54/75		2/30	Feces	MSP
(11)	Zhonghua	China	2015	20	7/10		0/10#	Tissue	qMSP
(12)	Wen-han	China	2015	178	69/89		3/92	Tissue	MSP
(12)	Wen-han	China	2015	119	63/89		2/30	Feces	MSP
(13)	Chao	China	2016	16	10/10		0/6	Tissue	BSP
(13)	Chao	China	2016	16	10/10		0/6	Feces	BSP
(14)	Heiying	China	2021	279	49/62	36/71	19/146	Feces	qMSP

AP, both adenomas and polyps; qMSP, quantitative methylation-specific PCR; MSP, methylation-specific polymerase chain reaction; BSP, bisulfite sequencing PCR.

#Adjacent non-cancerous.

Meta-analysis software package was used with Review Manager Version 4.2 (RMV4.2). The odds ratios (ORs) and 95% CIs were calculated using the statistical analysis of RMV4.2. The heterogeneity was insignificant if *p* > 0.05; the fixed-effects model in RMV4.2 was adopted; otherwise, the random-effects model in RMV4.2 was adopted. If OR > 1, and the upper and lower limits of the interval 95% CIs were all greater than 1, there is a high risk. The more the lower value of the interval including OR exceeded 1, the riskier it was. Publication bias was assessed with forest plot in RMV4.2. When all discrete points were within the 95% region of a forest plot, and they were centralized symmetrically, there was no publication bias in RMV4.2. Otherwise, if there was bias, it was evaluated according to the literature (15). All hypothesis tests concluding the differences of incidence frequency were evaluated with a 2-sided hypothesis test, and *p* < 0.01, based on statistical theory, indicated a significant difference. The curve fitting method, correlation study, and association study of the quadratic equation were calculated with SPSS Statistics 17.0 software. According to the mathematical statistical theory, the correct conclusions were given.

## Results

**Figure 1:**
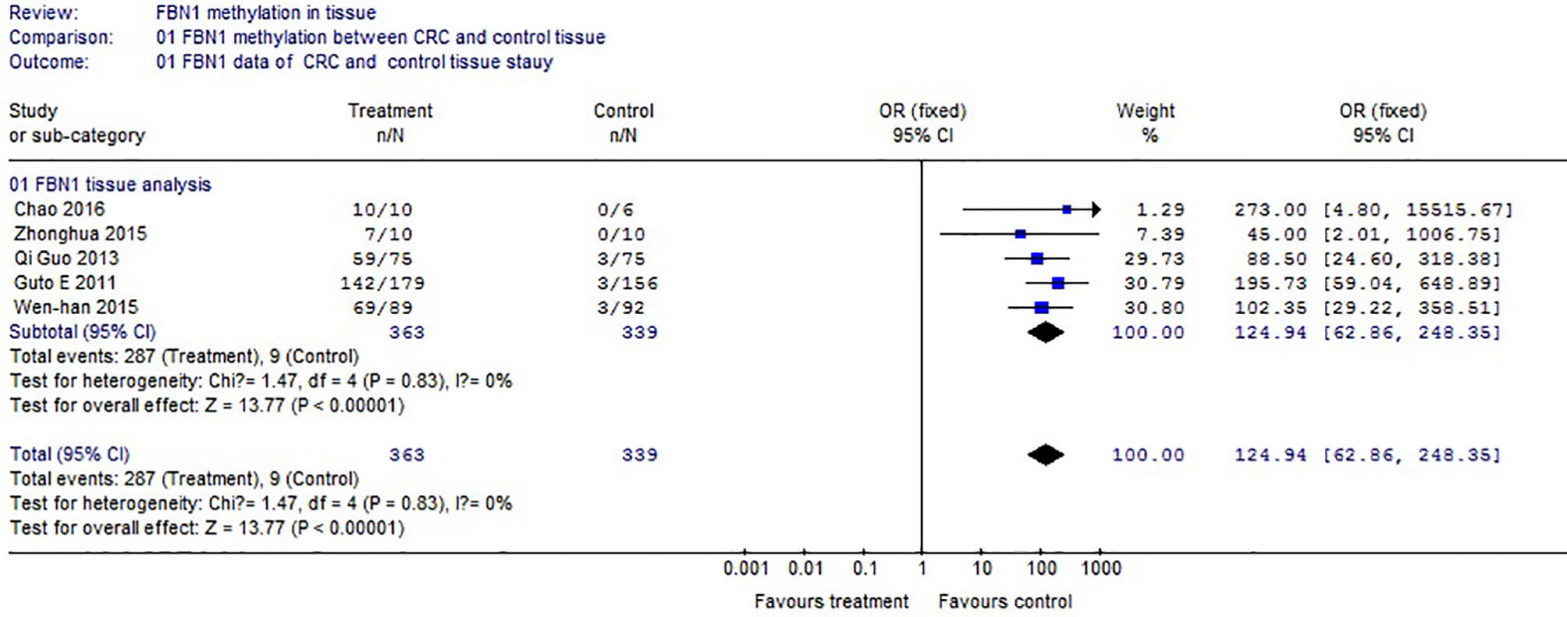
Forest plot of colorectal cancer and control methylation in FBN1 tissue.

**Figure 2:**
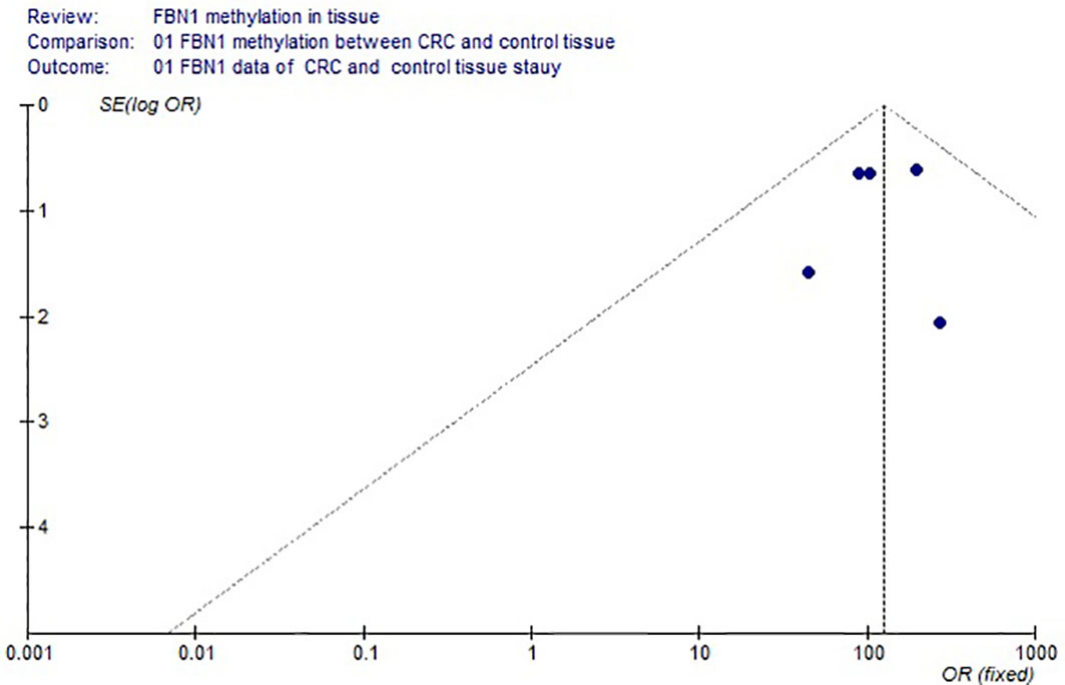
Funnel plot of colorectal cancer and control methylation in tissue.

**Figure 3:**
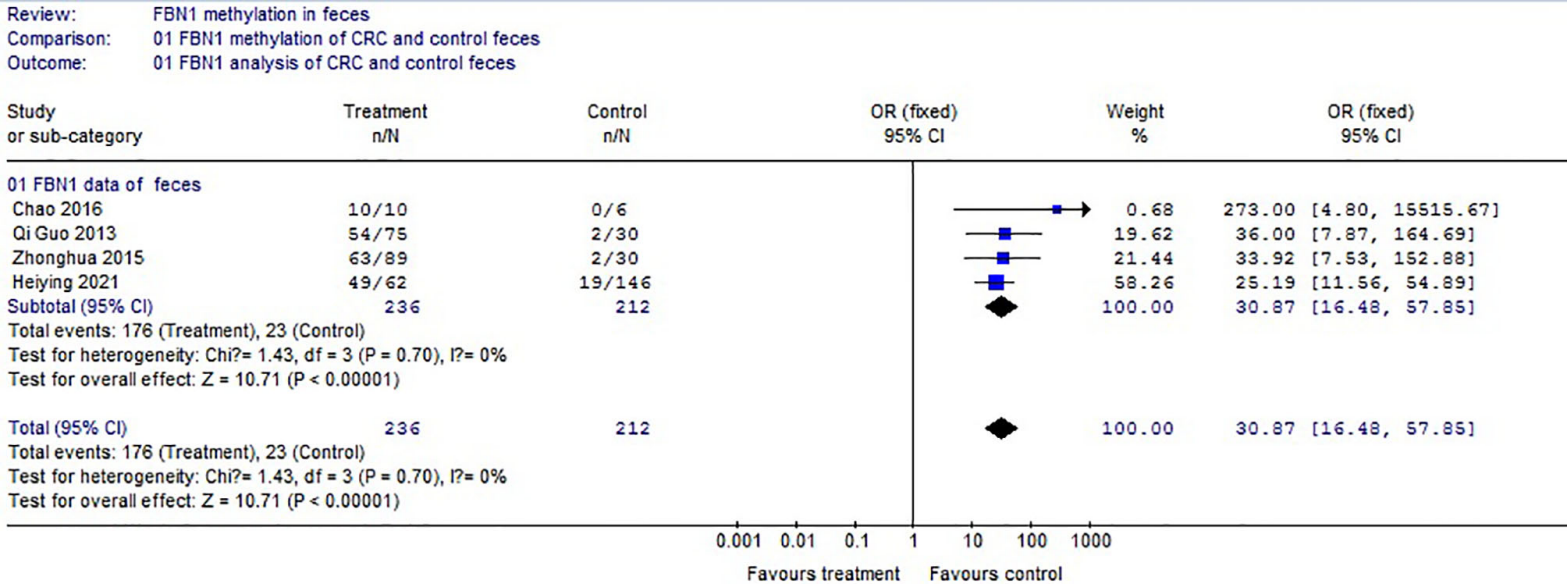
Forest plot of both adenomas or polyps and control methylation in FBN1 feces.

**Figure 4:**
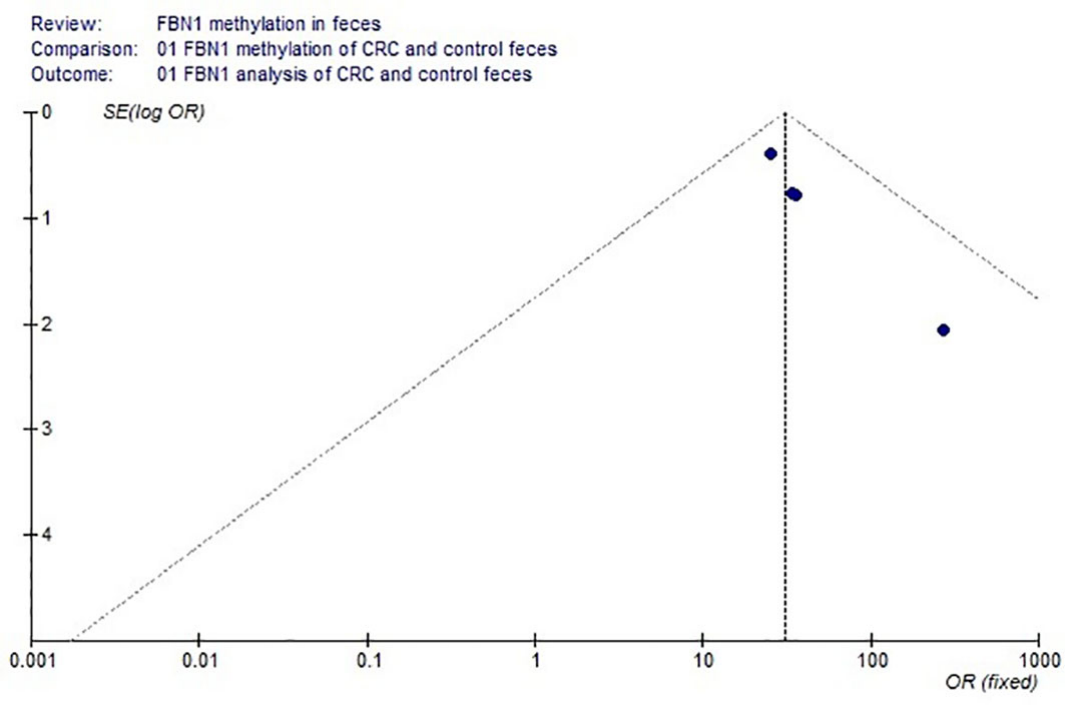
Funnel plot of both adenomas or polyps and control methylation in FBN1 feces.

Based on the systematic search method above, we had identified the 6 independent studies on FBN1 methylation in both CRC control study and adenoma or polyp control study, including 702 tissue and 448 fecal samples in **Table 1**. Because our meta-analysis can be satisfied independent of studies being performed under the same conditions, we can analyze the DNA methylation of tissue colorectal cells and that of cell-free DNA in feces. In tissue colorectal cells, total data including 363 CRCs and 339 controls in tissues in **Table 1** were calculated with Meta-analysis software. The calculating results are shown in **Figures 1** and **2**. Because *p* = 0.83 in **Figure 1**, the fixed-effects model was adopted. The analysis indicated that FBN1 methylation risk of CRC was significantly higher than that of controls [OR (95% CI) = 124.94 (62.86–248.35) and *p* < 0.00001; **Figure 1**]. Because 100% (5/5) of the scattered points fell within the 95% confidence region of the funnel plot in **Figure 2**, and the scattered points were symmetrical, there was no publication bias; therefore, the methylation of FBN1 in tissue was associated with CRC. In cell-free DNA in feces, according to the sample data including 236 samples with CRC and 212 samples with control in **Table 1**, the sample data were calculated with Meta-analysis software; this result had shown that because *p* = 0.70 in **Figure 3**, the fixed-effects model was adopted, and that because 100% (4/4) of the scattered points fell within the 95% confidence region of funnel plot in **Figure 4**, the scattered points were relatively symmetrical; the funnel plot in **Figure 4** proved no publication bias. The result had shown that there was a significantly high risk in CRC and control feces studies [OR (95% CI) = 30.87 (16.48–57.85), and *p* < 0.00001, in **Figure 3**].

**Figure 5:**
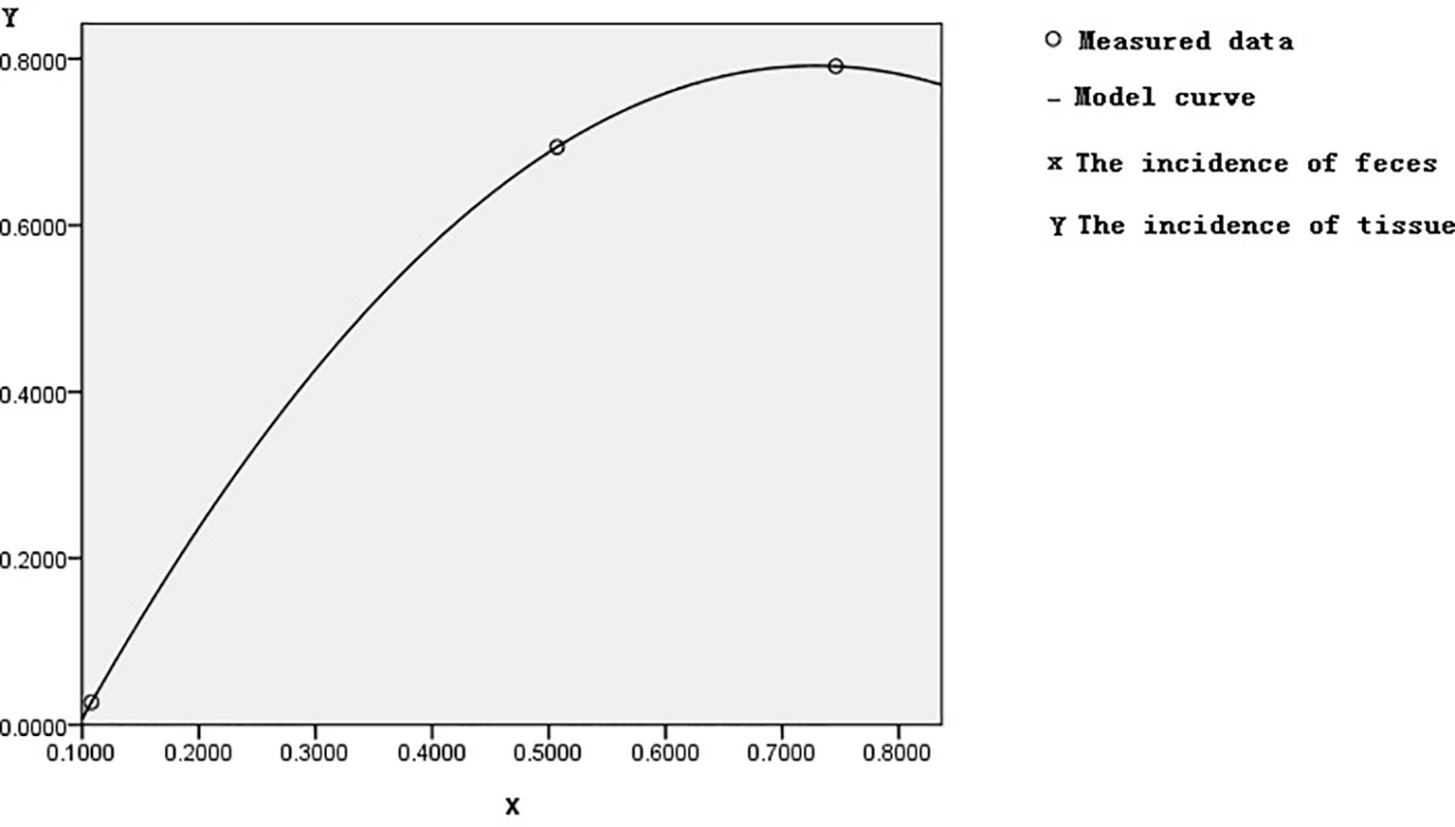
Quadratic graph of the incidence of methylation in tissue and feces.

Based on control feces as a standard reference in **Table 1**, the incidence of control methylation was 23/212 = 10.8%, corresponding to the incidences of both adenoma or polyp and CRC methylation, which were 36/71 = 50.7% and 176/236 = 74.6%. The incidences of 10.8%, 50.7%, and 74.6% of FBN1 methylation from healthy control to CRC though adenoma or polyp in feces were gradually increasing; this increasing risk size was closely related to the histological process of CRC evolution. In the samples of tissue colorectal cells, we used similar methods as above; the incidence of FBN1 methylation in the corresponding control, adenoma or polyp, and CRC was 2%, 69.4%, and 79.1%, respectively. The fecal incidence was taken as a transverse coordinate and represented by Feces M (Feces Methylation); the tissue incidence was taken as an ordinate coordinate and represented by Tissue M (Tissue Methylation). The curve fitting and association study were adopted by SPSS software. The quadratic curve equation (Tissue M) = −1.984 × (Feces M)^2^ + 2.892 × (Feces M) − 0.262 was obtained, where the correlation coefficient R was 1, and the corresponding Sig was 0. This model had statistical significance through correlation study. The graph of this quadratic curve equation is shown in **Figure 5**. According to both the quadratic curve equation and its graph, our discoveries indicated that feces methylation was highly associated with tissue methylation and might predict the incidence of methylation in tissues.

**Figure 6:**
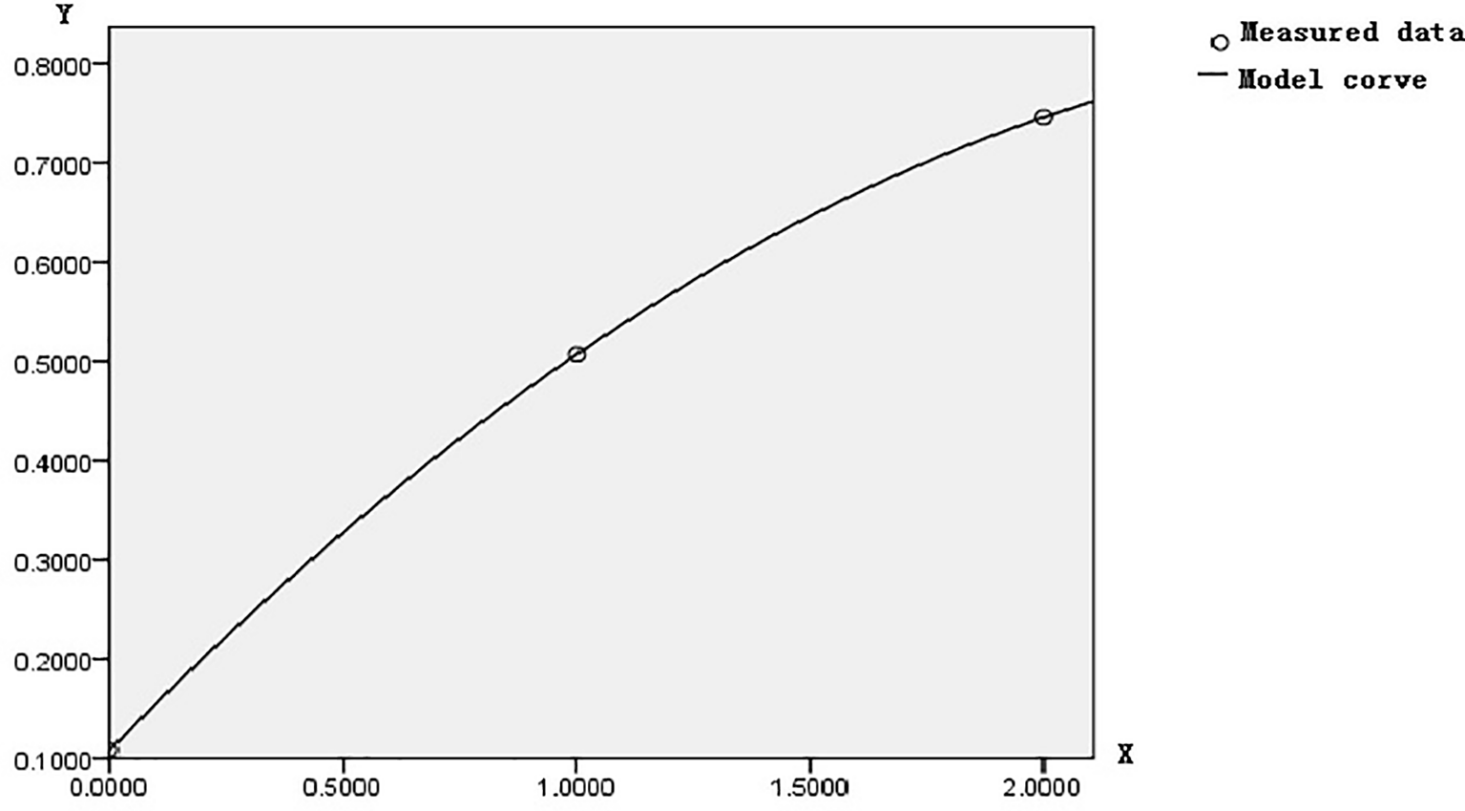
The quadratic graph of methylation incidence in the lesion tissue.

Let us set the tumor tissue evolution process as the abscissa X, where the normal control tissue is zero, the benign disease tissue is 1, and the CRC tissue is 2. Let us set their corresponding methylation incidence of 23/212, 36/71, and 176/236 as the ordinate Y; the optimal curve fitting equation calculated by SPSS 17.0 showed that the quadratic equation Y = 0.108 + 0.479X − 0.08X^2^ was significant. Where the correlation coefficient R = 1, the residual error was zero, and a small probability equals 0. Its corresponding figure is shown in **Figure 6**. The quadratic equation reflected the overall changing regulation of FBN1 methylation incidence in CRC lesions site during CRC development. Therefore, this overall changing regulation was gradually enhanced, promoted the transcriptional inactivation to gradually accelerate the expansion, and ultimately led to the occurrence of CRC.

## Discussions

Methylation of FBN1 associated with CRC had previously been reported (8). However, FBN1 methylation risk from control to CRC had not been reported through larger samples with meta-analysis; the risk size of FBN1 methylation still remained unclear. We firstly used meta-analysis to explore the risk size of FBN1 methylation. These results had proved that the OR of the colorectal cell tissues was 124.94 and that the OR of the cell-free DNA in feces was 30.87 in control and CRC studies. From a large sample point of view, the risk size of FBN1 methylation in tissues was up to 124.94; it was higher than the FBN1 methylation risk in previously published articles (8, 10–12). It had clearly proved that FBN1 methylation in tissue is an important and promising biomarker for identifying CRC. In addition, from the perspective of prevention or for people with a fear of colonoscopy, particular attention was paid to ensure no damage during testing. In no-injury feces detection, FBN1 methylation risk size was up to 30.87; this higher risk had illustrated that FBN1 methylation is a no-injury fecal biomarker in clinically early CRC diagnosis and screening. These risk size of FBN1 methylation was closely associated with the occurrence and biological development of CRC.

We had for the first time studied FBN1 methylation risk from controls to CRC through adenoma or polyp. We had found that FBN1 methylation incidence of control, adenoma or polyp patients, and CRC in tissue was 2.7%, 69.4%, and 79.1%, respectively, and that in feces it was 10.8%, 50.7%, and 74.6%, respectively. The results had illustrated that FBN1 methylation risk in tissue was significantly higher than that in feces. In addition, on the basis of corresponding the incidence risk of FBN1 methylation in feces to that in tissues, there was a quadratic curve equation (Tissue M) = −1.984 × (Feces M)^2^ + 2.892 × (Feces M) − 0.262. The discoveries indicated that feces methylation was highly associated with tissue methylation, the methylation of cell-free DNA in feces reflected that of colorectal cells in tissues, and the risk sizes of FBN1 methylation from normal control to CRC though adenoma or polyp were gradually increased in colorectal tissues and feces detection. Through the test results of the experimental methylation of human feces, using the above equation, we can calculate the FBN1 methylation risk size of the human mass; the accurate assessment of tumor risk was achieved in no-injury detection.

Because FBN1 methylation incidence of individual normal people was 2.7% in tissue and 10.8% in feces as seen in **Table 1**, these FBN1 methylation abnormalities can show that the mechanism of CRC occurrence begins with normal colonic epithelium under some certain environment. Because methylation regulation was one of the important epigenetic regulators, and thus the epigenetic regulation of methylation was closely related to the transcriptional regulation of tumor-related genes (16, 17), the transcriptional inactivation associated with FBN1 methylation might start from the normal colonic epithelium.

CRC develops through an ordered series of events beginning with the transformation of normal colonic epithelium to an adenomatous intermediate and then ultimately adenocarcinoma (18, 19). This variation of CIMP pathways should be followed during tumor evolution progression. Our study revealed that the incidence of FBN1 methylation from control to CRC though adenoma or polyp can gradually produce powerful strength according to the quadratic equation Y = 0.108 + 0.479X − 0.08X^2^; the transcriptional inactivation associated with FBN1 methylation might start from normal colonic epithelium; the FBN1 methylation incidence related with the quadratic equation promoted the transcriptional inactivation to gradually accelerate the expansion and ultimately lead to the occurrence of CRC.

Because DNA methylation was catalyzed and maintained by DNMT, DNA methylation in tumor development embodied the role of tumor-related DNMT (16, 17). Based on this quadratic curve equation, the metabolizing speed of the DNMT that can catalyze FBN1 methylation might begin slowly, then gradually increase during benign diseases (adenoma or polyp), and then rapidly develop during CRC; this process of DNMT synthesizes or degrades physiological activity and participates in the transformation or progression of human cancers by mediating the methylation of cancer suppressors. DNMT, which affected FBN1 methylation by DNMT, produced the silencing of tumor suppressor miRNA-encoding genes and directly affected carcinogenesis.

The fecal detection incurs no damage; it will have a broad application prospect, especially in clinical routine. The methylation of many genes was shown to be associated with CRC. The combination of several gene methylations will be the current direction in detection. The study of CIMP had enhanced the sensitivity for cancer recurrence monitoring; people are looking for better indicators of CIMP to improve the accuracy of early screening and diagnosis of CRC, which is also the direction of future development (20, 21). However, to achieve the clinical application, a large number of problems will be solved, for example, class, gender, age, and ethnicity, fewer samples, and the impact of tumor staging, and so on (22–24); they all need to be further studied. Because the molecular mechanism and the variation of many factors of CRC are very complicated, and the clinical symptoms are very hard to detect (24, 25), it will take a long time to solve some problems of screening and early diagnosis of CRC.

## Conclusions

The whole process of methylation pathogenesis during CRC development is discovered that the transcriptional inactivation associated with FBN1 methylation might start from the normal colonic epithelium and can gradually enhance, accelerate the expansion, and ultimately lead to the occurrence of CRC. The overall process of DNMT changing also has the feature of the quadratic curve of FBN1 methylation and plays a role in DNMT mechanism. FBN1 methylation is an important biomarker based on the studies of large experimental data. The risk size of fecal methylation can accurately predict that of tissue methylation in non-invasive detection.

## Data Availability Statement

The datasets presented in this study can be found in online repositories. The names of the repository/repositories and accession number(s) can be found in the article/supplementary material.

## Ethics Statement

Ethical review and approval were not required for the study on human participants in accordance with the local legislation and institutional requirements. Written informed consent for participation was not required for this study in accordance with the national legislation and the institutional requirements.

## Author Contributions

LL, GW, and JM acquired the data and wrote the text. The other authors have made a lot of contributions.

## Conflict of Interest

The authors declare that the research was conducted in the absence of any commercial or financial relationships that could be construed as a potential conflict of interest.

## Publisher’s Note

All claims expressed in this article are solely those of the authors and do not necessarily represent those of their affiliated organizations, or those of the publisher, the editors and the reviewers. Any product that may be evaluated in this article, or claim that may be made by its manufacturer, is not guaranteed or endorsed by the publisher.
